# High Rate of Awarding Compensation for Claims of Injuries Related to Clinical Trials by Pharmaceutical Companies in Japan: A Questionnaire Survey

**DOI:** 10.1371/journal.pone.0084998

**Published:** 2014-01-08

**Authors:** Chieko Kurihara, Hideo Kusuoka, Shunsuke Ono, Naoko Kakee, Kazuyuki Saito, Kenji Takehara, Kiyokazu Tsujide, Yuzo Nabeoka, Takuya Sakuhiro, Hiroshi Aoki, Noriko Morishita, Chieko Suzuki, Shigeo Kachi, Emiko Kondo, Yukiko Komori, Tetsu Isobe, Shigeru Kageyama, Hiroshi Watanabe

**Affiliations:** 1 Molecular Imaging Center, National Institute of Radiological Sciences, Chiba, Chiba, Japan; 2 National Hospital Organization Osaka National Hospital, Osaka, Osaka, Japan; 3 Graduate School of Pharmaceutical Sciences, The University of Tokyo, Tokyo, Japan; 4 Department of Health Policy, National Center for Child Health and Development, Tokyo, Japan; 5 Office of OTC/Generic Drugs, Pharmaceuticals and Medical Devices Agency, Tokyo, Japan; 6 Clinical Evaluation Expert Committee, Drug Evaluation Committee, Japan Pharmaceutical Manufacturers Association, Tokyo, Japan; 7 Clinical Research Coordination Department, Chugai Pharmaceutical Co., Ltd., Tokyo, Japan; 8 Development Division, Mitsubishi Tanabe Pharma Corporation, Tokyo, Japan; 9 Administration Center for Clinical Trials Institute for Clinical Research, National Hospital Organization Osaka National Hospital, Osaka, Osaka, Japan; 10 Center for Clinical Research, Hamamatsu University School of Medicine, Hamamatsu, Hamamatsu, Japan; 11 Office of Conformity Audit, Pharmaceuticals and Medical Devices Agency, Tokyo, Japan; 12 Office of New Drug III (CNS/PNS/Anesthesia), Pharmaceuticals and Medical Devices Agency, Tokyo, Japan; 13 Keio Law School, Keio University, Tokyo, Japan; 14 Division of Clinical Pharmacology and Therapeutics, The Jikei University School of Medicine, Tokyo, Japan; 15 Department of Clinical Pharmacology and Therapeutics, Hamamatsu University School of Medicine, Hamamatsu, Hamamatsu, Japan; The George Washington University Medical Center, United States of America

## Abstract

**Introduction:**

International norms and ethical standards have suggested that compensation for research-related injury should be provided to injured research volunteers. However, statistical data of incidence of compensation claims and the rate of awarding them have been rarely reported.

**Method:**

Questionnaire surveys were sent to pharmaceutical companies and medical institutions, focusing on industry-initiated clinical trials aiming at new drug applications (NDAs) on patient volunteers in Japan.

**Results:**

With the answers from pharmaceutical companies, the incidence of compensation was 0.8%, including 0.06% of monetary compensation. Of the cases of compensation claims, 99% were awarded. In turn, with the answers from medical institutions, the incidence of compensation was 0.6%, including 0.4% of serious but not death cases, and 0.04% of death cases. Furthermore, most claims for compensation were initiated by medical institutions, rather than by the patients. On the other hand, with the answers from clinical trial volunteers, 3% of respondents received compensations. These compensated cases were 25% of the injuries which cannot be ruled out from the scope of compensation.

**Conclusion:**

Our study results demonstrated that Japanese pharmaceutical companies have provided a high rate of compensation for clinical trial-related injuries despite the possibility of overestimation. In the era of global clinical development, our study indicates the importance of further surveys to find each country's compensation policy by determining how it is being implemented based on a survey of the actual status of compensation coming from statistical data.

## Introduction

International norms and ethical standards on clinical research have suggested that compensation for research-related injury should be provided to injured research volunteers [1]–[5]. However, statistical data of incidence of compensation claims and the rate of awarding them have been rarely and insufficiently reported [6]–[15]. The aim of this article is to report the results of our survey to find the rate of awarding compensation claims for injuries of patient volunteers in clinical trials by pharmaceutical companies in Japan.

Generally, in civilized countries civil law assures the citizens of the right to claim compensation for damage caused by negligence, and in this case a person who claims has to prove the negligence and causal relationship between the cause and the damage. In such countries that have a comprehensive no-fault compensation framework in the area of medical care, patients who claim do not have to prove causality, but when compensation is granted, their claims against negligence liability may be limited. In such countries that do not have a comprehensive no-fault compensation framework, some specific laws may define compensation frameworks in specific areas.

Some of the European countries have a national framework that provides medical care and compensation for medical-related injuries including those in clinical research [13], [16], [17], [18]. This includes payment of the medical cost for the treatment of the injuries as well as monetary compensation in cases of death or permanent disability. In the United States (U. S.) and most of the developing countries, medical care is provided through mixed sources, from public and private sectors, and there is no assurance that research-related injuries will be compensated [13].

In Japan, approximately 70% of the cost of medical care is covered by public health insurance, while 30% is paid by patients. Compensation frameworks in medical area are limited to “Relief System for Adverse Health Effects”, including “Adverse Drug Reaction (ADR) Relief System” which covers compensation for ADRs of marketed drugs; and other specific limited areas. Good Clinical Practice (GCP) Ordinance [19] under the Pharmaceutical Affairs Law, which covers clinical trials aiming at new drug applications (NDAs) and legally conditioned post-marketing trials, defines the sponsor's responsibility to provide compensation and not to burden injured volunteers to prove causality. Ethical guidelines covering clinical research [20] not aiming at NDAs briefly define the responsibility of investigators to provide compensation.

Detailed compensation policies are not defined in governmental regulations but defined in the Guidelines by The Japan Pharmaceutical Industry Legal Affairs Association (JPILA) [21]. According to the guidelines, 30% (patients' co-pay) of the cost of treatment for research-related injury should be paid by the sponsor of the clinical trial as a part of compensation. Also monetary compensation for death or disability should be provided by the sponsor.

JPILA guidelines were developed in 1999 and revised in 2009 [21], using examples from the guidelines by the Association of British Pharmaceutical Industries (ABPI) issued in 1991 [22], which cover clinical trials involving patient volunteers (Table S1). ABPI issued other guidelines separately for healthy volunteers [23]–[25]. JPILA guidelines suggest that the amount of compensation should be calculated based on the “ADR Relief System” in the cases of patient volunteers; or based on “Workers' Accident Compensation Liability Insurance” or “Relief Service for Injury to Health with Vaccination” in the cases of healthy volunteers.

This article describes the results of our survey of awarding compensation claims in clinical trials conducted by Japanese pharmaceutical companies, according to the above mentioned regulatory framework and compensation policy by JPILA.

## Methods

### Questionnaire surveys

Our questionnaire surveys were conducted as follows:

(1) We delivered questionnaire sheets by postal mail to 68 companies, members of the Japan Pharmaceutical Manufacturers Association (JPMA), and asked about the clinical trials aiming at NDAs, which were completed in the period from April 2009 to March 2010, excluding phase 1 trials on healthy volunteers.

(2) We introduced a questionnaire using a web-system, to the 194 medical institutions, the members of the National Hospital Organization, and the others engaged in three categories of public funding projects; to the 43 Site Management Organizations (SMOs); and to the Clinical Research Coordinators (CRCs) belonging to these institutions. We asked about the clinical trials which they were engaged in during the period from April 2007 to October 2010. Most of these clinical trials conducted in hospitals are supposed to be ones involving patient volunteers.

(3) We provided 206 questionnaire sheets through 4 medical institutions of some of the authors to be delivered during the period of January and/or February of 2011 to study volunteers (who were or are hospitalized or receiving medical attention as research participants) or their representatives.

The items and constructions of the questionnaires were not the same for the three target groups (companies, hospitals, volunteers) in terms of the feasibility of obtaining survey data from them.

### Ethics committee review

All of the answers were provided anonymously, and we did not deal with individual identifiable information; therefore, ethics committee approval was not required according to both the Declaration of Helsinki [5] and Japanese governmental guidelines [20], but a part of the questionnaire survey on patients was approved by the ethics committee of National Center for Child Health and Development (an ethics committee inside of the national hospital, specialized in pediatrics).

## Results

### Response rates of questionnaire surveys

From the above questionnaire surveys, the answers were obtained from: (1) 44 of the 68 member companies of the JPMA (response rate: 65%); (2) 86 of 194 medical institutions and 28 of 43 Site Management Organizations (SMOs) to which this questionnaire was introduced, and more voluntary participating ones; and 769 CRCs who belong to these institutions or SMOs (we do not mention response rate because we do not know how many institutions and individual CRCs had the chance of answering this part of the survey distributed by a web-based system); and (3) 115 research volunteers or their representatives among those whom 206 questionnaires sheets were delivered in 4 hospitals (response rate: 56%)

### Incidence and contents of compensation

The numbers of the cases and the contents of compensations found from the questionnaire survey results are summarized in **Table 1**.

**Table 1:** Incidence and contents of compensation cases of industry-initiated clinical trials in Japan.

	Sponsor companies	Medical institutions	Volunteers
The surveyed population	68 member companies of JPMA.	237 institutions. (introduced to 194 hospitals and 43 SMOs, but more institutions may have been able to access the survey.)	206 questionnaires sheets were delivered in 4 hospitals to volunteers or their representatives.
Respondents to the survey	44 companies. (Response rate: 65%)	114 institutions. (86 hospitals and 28 SMOs)	115 volunteers or their representatives. (Response rate: 56%)
		(Response rate is unknown, because of the web-based survey)	
Terms to be surveyed	Protocols except phase 1 on healthy volunteers which were completed in one year from April 2009 to March 2010.	Protocols covering phase 1 to 3 conducted in three years from April 2007 to October 2010.	Volunteers or their representatives, who were visiting institutions or were hospitalized in January and February 2011.
Numbers of protocols	183	Approximately 40 protocols in each institutions and approximately 26 protocols in each SMO* ^1^.	Not surveyed.
Numbers of participants in surveyed clinical trials	32,318	21,065	115 (respondents)
Cases of injuries	Not surveyed.	Not surveyed.	19 (17% of the respondents, 17/19 described the contents of injuries)
Claims for compensations or cases which may be within the scope of compensations	Claims for compensation: 250 (0.8% of participants, there were 251 claims but 1 subject withdrew.)	Not surveyed.	Cases some of which may be within the scope of compensation: 12 (10% of the respondents: 3/12 compensated; 9/12 not compensated but cannot be ruled out from the scope of compensation, including 4/9 gave a reason and 5/9 did not give any reason.)
Compensated cases	249 cases, including 2 cases compensated on being proposed by companies without claims from subjects. (0.8% of the participants; 247/250 = 99% rate of awarding the claims)	132 cases. (0.6% of the participants)	3 cases. (3/115, 3% of the respondents; 3/19, 16% of the injury cases; 3/12, 25% of the cases which cannot be ruled out)
			
Not compensated cases	Totally 4 cases. (3 cases were not compensated; 1 case withdrew.)	Not surveyed.	Totally 16 cases. (9/16 cannot be ruled out, including 5 without the reason given; and 7/16 outside the scope, including 5without medical cost, 2 not related to test drugs).
Details of the contents of compensated cases (%: among the participants)	For 229 cases (0.7%), only medical cost was paid; For 20 cases (0.06%), not only medical cost, but also monetary compensations were paid.	For 38 less serious cases (0.18%) and for 84 hospitalization or more serious but not fatal cases (0.4%), only medical cost was paid; For 9 cases of death (0.04%), monetary compensations were paid for bereaved families in addition to medical cost.	Not surveyed.

1 As many of the same protocols are conducted in multiple institutions, we cannot determine the total number of protocols. Reconstructed from the survey result reported in the reference No. 12.

#### (1) Survey of companies

As for the survey of companies, the 44 companies who responded to our survey completed 183 protocols, excluding phase 1 studies on healthy volunteers, in the period from April 2009 to March 2010. A total of 32,318 patient volunteers participated, and there were 251 claims for compensations and 1 case withdrew the claim. Among these 250 claims (0.8% of the total participants), 247 cases were compensated (99% of the cases of claims were compensated). Additionally, for 2 cases without claims from patients, companies proposed to provide compensations. Thereby, 249 compensations were awarded (0.8% of the total participants). We did not survey the reasons why three cases of claims were rejected.

The contents of the 249 cases of compensations are as follows: for 229 cases, only “medical expenses” (which means that companies have paid 30% of the total medical costs, which were to be paid by patients if there was no compensation from companies;, whereas 70% is to be paid from public insurance) or both the “medical expenses” and “medical allowance” (for miscellaneous expenditures such as transport expenses, incidental costs, etc.) were paid (92% of the total compensation cases, 0.7% of the total study participants); for 20 cases, not only medical expenses and medical allowance, but also monetary compensations were paid (8% of the total compensation cases, 0.06% of the total study participants).

#### (2) Survey of medical institutions

As for the survey of medical institutions, at the 86 medical institutions as well as 28 SMOs who responded to our survey, approximately 40 protocols in each institution or approximately 26 protocols in each SMO covering phase 1 to 3 were conducted in three and a half years from April 2007 to October 2010, and 21,065 volunteers participated. A total of 132 cases were compensated (0.6% of the total participants). The contents of 132 compensation cases are as follows: 38 less serious cases, where medical expenses and/or medical allowance were paid (0.18% of the total participants); 84 hospitalizations or more serious but not fatal cases, where medical expenses and/or medical allowance were paid (0.4% of the total participants); and 9 death cases where monetary compensations were paid for bereaved families in addition to medical expenses and/or medical allowance were paid (0.04% of the total participants).

In this part of our survey, numbers of actual cases of injuries and of claims for compensations were not obtained, but as described later, to introduce the survey results on the proposal of compensations, there was at least 1 case of a claim which was not compensated because it was regarded not to be caused by the investigational product.

As a part of the survey of medical institutions, we asked who proposed the claims for compensations, and the answers were obtained from hospitals, SMOs, CRCs as shown in the Figure S1 (for CRCs, multiple answers were allowed). We found that claims for compensations were proposed from the side of the medical institutions rather than from the side of research volunteers. Sixteen percent of the answers by CRCs stated that proposals of compensation were from the side of companies (though some of the CRCs may refer to the same cases).

On the other hand, there was at least 1 case of a claim which was not compensated because it was regarded not to be caused by the investigational product.

#### (3) Survey of volunteers

As for the survey of clinical trial volunteers, 115 volunteers or their representatives, who were visiting institutions or were hospitalized in January and/or February of 2011, responded to our survey.

Nineteen of 115 answered that they had some experiences of injury (17% of the respondents), of which 3 cases were compensated (3% of the respondents). Seventeen of the 19 described their injuries, most of which did not seem to be serious, and a few which may have been serious but not life-threatening. Among the 17 cases who described their injuries, 9 cases participated in the studies on anticancer drugs. Among the 16 who experienced injuries but were not compensated, 11 cases described the reasons why they were not compensated. Five cases said “there was no medical cost” and 2 cases said that there was “no relation with the investigational drug”. These 7 cases seem to be outside the scope of compensation. The other 4 cases described the reasons: “I myself paid my own medical cost”; I have not confirmed how the situation was”; “Such less serious cases would be inevitable”; “Causality with the tested drug is unclear and the injury is not severe and the case is now in follow-up”. Some of these 4 cases who gave reasons and the 5 cases who did not give the reason (totally 9) may be within the scope of compensation but were not compensated, which means cannot be ruled out. Therefore, 3 of 115 respondents (3%); 3 of 19 injury cases (16%); or 3 of the 12 which cannot be ruled out (25%) were compensated. This compensation rate was much different from the one obtained from the survey of pharmaceutical companies. However, overall, there was no answer expressing an experience in which they claimed for compensation but failed to get it.

We also asked the volunteers about their understanding and satisfaction about the compensation. Ninety-one of 115 (79% of the respondents) answered that they knew that they can claim for compensation for research-related injuries. Sixty-five (57% of the respondents) answered that they received an explanation about compensation and 35 (30%) answered that they were not sure whether they did receive one. Eighteen of 65 who received an explanation (16% of the respondents) answered they could clearly understand the explanation and 42 (37%) answered that they mostly understood.

We asked three volunteers who were compensated about their impression and all of them seem to be satisfied with procedures, but one of the three was not satisfied with the content of the compensation (Table S2). Among the three compensation claims, one was made from a volunteer and the other two were from a doctor or a CRC.

## Discussion

Here we discuss the implications of our research results from perspectives of (1) data analysis of our survey; (2) comparison with other surveys; and (3) ethical considerations.

### Data analysis of our survey

Our survey results suggest that Japanese companies have a high rate of awarding compensations, but this may come from overestimating the results. There is a possibility that some companies who experienced problematic cases did not respond to our survey. Additionally, the survey of volunteers suggests that 12 volunteers cannot be ruled out from the scope of compensation but only 3 were compensated. We could not find any information from all of the volunteer's answers that there were some cases that they claimed but were not awarded. However, there may be such cases in which they did not claim even if they experienced injury which can be compensated; or other cases in which they withdrew their claims through consultation with research staff (especially in such cases when the judgment of causality is difficult). We also found that in 1 of the 3 compensated cases, the recipient thought that the provided compensation did not match the level of the injury. Therefore, we found that companies' compensation rate was 99% but some of these compensated volunteers may feel that it did not match the level of injury.

### Comparison with other surveys

Previously to this survey, JPILA conducted a survey of rate of awarding compensation claims of affiliated companies' clinical trials for the 5-year period since 2003 [26]. Among 763 claims, 730 (96%) were awarded (678 were for medical cost). These 33 were outside the scope of the guidelines and among these 33, a causal relationship was ruled out in 18.

The Japanese government reported compensation rates of awarding claims mainly focusing on the cases caused by the use of drugs as follows: 28% in Sweden; 25% in Denmark; 23% in Norway; 42% in Finland; 46% in France; and 66% in New Zealand; and in Japan 88% [18]. The periods of the data collections vary among these countries. Among these four Nordic countries, the data includes the cases of clinical trials, but the rates specific to clinical trials have not been obtained. Although the background of these data varies among the countries, the Japanese government seems to provide a relatively high rate of awarding compensation. This Japanese data does not include compensation cases in clinical trials under the Pharmaceutical Affairs Law, which is outside the scope of “ADR Relief System”.

On the other hand, reports from U. S. and India [27]–[29] found that 22–91% of the informed consent documents (ICDs) of clinical research studies (rates vary according to sub-categories of research) which were available through web-sites stated to provide free treatment for research-related injuries. In the U. S. 72% of these available ICDs stated that they could not provide monetary compensation for death or disability [27]. In India, such monetary compensation was very rarely assured in ICDs [28]. A report from South Africa [14] found that there were claims for compensation from a clinical trial which was suspended based on data from an international companion study that indicated no evidence of efficacy and greater risk of harm. Another report from India found that, although GCP regulations require compensation for trial-related injuries, in some clinical trials only five families of 25 trial-related death cases had received monetary compensation. However, later after being instructed by the regulatory authority, 17 additional families (total of 22/25) received compensation [15]. Responding to such a situation, the Indian government issued guidelines for determining the amount of financial compensation and in 2013 they included these guidelines in the Drugs and Cosmetic Rules [30].

### Ethical considerations

Despite the possibility of overestimation, we suppose that the reason of the high rate of awarding compensation by Japanese companies may be because of the instructions of JPILA guidelines which define detailed procedures of calculating the amount of compensation (Table S1). The guidelines also suggest that the investigator should provide a written document to outline the company's compensation policies and explain it to the volunteers at the time of obtaining informed consent. Also our survey found that there were several cases in which medical institutions or companies made proposals of compensation even if volunteers did not make a claim.

ABPI guidelines for patient volunteers suggest that amount of compensation should be defined consistent with the amount commonly awarded for similar injuries by English Court in cases of legal liability but they do not suggest the amounts according to the severities of injuries. ABPI also suggest that the copies of the guidelines should be provided to the volunteers according to their requests, but they do not recommend that the copies should be handed to volunteers prior to obtaining informed consent. ABPI guidelines have been adopted as industrial policies in Australia [31], in New Zealand [32]; used as a part of national guidelines in South Africa [33]; and in Singapore [34]. We have not yet found any statistical data concerning how these guidelines have been implemented along with the governmental regulations.

The policies of JPILA and the attitudes of Japanese companies seem to be desirable from the standpoint of the ethical principle “respect for persons (informed consent)” and “beneficence (maximization of benefit)” advocated in the Belmont Report [35]. On the other hand, from the standpoint of “justice (fairness in distribution of benefit)”, other difficult questions are raised. When Japanese companies conduct clinical trials in other countries where the standards of compensation are not as generous as the standards in Japan, which standards have been actually followed, those of the host country or those of Japan? In particular, what are the common practices in the cases of a protocol for multi-national clinical trials? Is there not the possibility that such a high standard of awarding compensation might lead to clinical trials being conducted outside of Japan where the cost of compensation is lower than in Japan? This kind of issue of “justice” is especially critical in the era of global clinical development. Additionally, even among the clinical trials conducted in Japan, there is a discrepancy in the regulations between company-initiated and academic researcher-initiated trials. This may cause the unfair distribution of benefits even in the domestic research community.

## Conclusion

Our study results demonstrated that the Japanese pharmaceutical companies have provided a high rate of awarding compensation for claims of injuries related to clinical trials despite the possibility of overestimation. This survey was limited to the cases of industry-initiated clinical trials involving patient volunteers aiming at NDAs in Japan. While it is desirable to implement this kind of high standard of providing compensation for volunteers of any type of research everywhere in the world, at this time, we cannot promptly advocate that this Japanese policy should be universal since it is difficult to be implemented in a resource-poor setting. However, the direction of the road ahead is to realize a higher level of human subject protection that is just and fair throughout the world.

So we conclude that, in the era of multi-national clinical development, it is important to promote further surveys and international exchanges of information of each country's compensation policy for research-related injuries. This should include the actual status of how the policy is being implemented to award compensation based on statistical data of the incidence of injuries, compensation claims and awarded cases, and the contents of compensations actually granted.

## Supporting Information

Table S1 Comparison of policies between ABPI (Association of British Pharmaceutical Industries) and JPILA (Japan Pharmaceutical Industry Legal Affairs Association) clinical trial compensation guidelines^*1^ and inclusion of each item of policy in the internal guidelines of 12 Japanese companies^*2^ (Both ABPI and JPILA guidelines are not legally defined but industry's voluntary guidelines).
(DOCX)

Table S2 Expression and impression of the volunteers who received compensation.
(DOCX)

Figure S1 Persons to propose about compensation when research-related injuries occur.
(JPG)
