# Supplementary material for: High Rate of Awarding Compensation for Claims of Injuries Related to Clinical Trials by Pharmaceutical Companies in Japan: A Questionnaire Survey
Source: PLoS One. 2014 Jan 8;9(1):e84998. doi: 10.1371/journal.pone.0084998 (PMC3885663; doi:10.1371/journal.pone.0084998)
Supplement: Table S1 — Comparison of policies between ABPI (Association of British Pharmaceutical Industries) and JPILA (Japan Pharmaceutical Industry Legal Affairs Association) clinical trial compensation guidelines*1 and inclusion of each item of policy in the internal guidelines of 12 Japanese companies*2 (Both ABPI and JPILA guidelines are not legally defined but industry's voluntary guidelines). (DOCX) [file pone.0084998.s001.docx]

**Table S1.** Comparison of policies between ABPI (Association of British Pharmaceutical Industries) and JPILA (Japan Pharmaceutical Industry Legal Affairs Association) clinical trial compensation guidelines^*1^ and inclusion of each item of policy in the internal guidelines of 12 Japanese companies^*2^ (Both ABPI and JPILA guidelines are not legally defined but industry’s voluntary guidelines)

|  | ABPI guideline | JPILA guideline | Number of companies which include each item in their ICDs (n=12)*2 |
| --- | --- | --- | --- |
| **Responsibility and causality** |  |  |  |
| No fault compensation. | 〇Yes. (1.1) | 〇Yes. (1-1) | 10 |
| Injury resulting from the clinical trial (study drug or procedure defined in the protocol) could be compensated. | 〇Yes. (1.2) | 〇Yes. (1-3) | 12 |
| Study subject (volunteer) does not have to prove negligence and/or causality. | 〇Yes. (1.7) | 〇Yes. (1-1) | 7 |
| Agreement by company to abide by the guidelines does not affect the right of the volunteer pursue a legal remedy. | △Yes, but the volunteers will normally be asked to accept that any payment made under the Guidelines will be in full settlement of their claims. (5.3) | 〇Yes. (1-2) | 11 |
| **Range and limitation** |  |  |  |
| Compensation for a child injured in utero. | 〇Yes. (1.3) | －Not mentioned. | 0 |
| Limitation of compensation according to benefit/risk (severity of disease, probability of events), and consent of the volunteers. | △ Compensation may be limited according to benefit/risk and consent of the volunteer. (4) | △ Compensation may be limited according to benefit/risk and consent of the volunteer. (4-2) | 0 |
| Exclusion of specific classes of drugs. | －Not mentioned. | △Only medical cost is compensated in cases of carcinostatic substances, and immunosuppressive agents, but considering the benefit/risk, monetary compensation may be provided*3.(4-3-2) | (Not applicable, because it is defined in each study protocol.) |
| Injury caused by marketed drug. | ×Not covered by the guideline. (2.3) | ×Not covered by the guideline. (4-3-1) | (Not applicable, because it is defined in each study protocol.) |
| To fail to get therapeutic benefit due to receiving placebo. | ×Not compensated. (3.3) | △In principle not compensated, but may be compensated in specific cases, e.g. aggravation caused by placebo use during washout period. (3-2) | 9 |
| Failure of the study drug to have the intended effect. | ×Not compensated. (3.1) | △In principle not compensated, but may be considered whether or not to be compensated in specific cases, e.g., aggravation compared to the baseline which could not have occurred without the participation in the clinical trial. (3-1) | 12 |
| Injury caused by an incidental accident, not due to the administration of the investigational drug. | ×－Not mentioned. | ×Not compensated. (2-1) | 11 |
| Injury resulting from significant departure from the protocol. | ×Not compensated. (3.4.1) | ×Injuries for which medical institution is responsible should not be compensated by the sponsor company as it is the liability of responsible person. (2-2) | 1 |
| Wrongful or negligent act of a third party, including a doctor’s failure to deal adequately with an injury. | ×Not compensated. (3.4.2) | ×Not compensated as it is the liability of the third party. (2-2,3) | 6 |
| Negligence of the volunteer. | △Not compensated or should be abated considering the level of contributory negligence of the volunteer. (3.4.3) | △Not compensated or should be abated considering the level of contributory negligence of the volunteer. (2-5, 3-3) | 12 |
| **Compensation according to severity of Injury** |  |  |  |
| Less serious, curable injury. | ×Not compensated in case of patient volunteer (1.4); For healthy volunteer~~s~~, any injury should be compensated (There are other guidelines specific to healthy volunteer). | 〇Only medical cost should be compensated, calculated based on other governmental system of compensating injuries*4. (4-1-1, 2) | 12 |
| Serious and/or irreversible injury or death. | 〇The amount of compensation should be appropriate to the nature, severity and persistence of the injury and should be consistent with the amount awarded for similar injuries by an English Court in cases of legal liability. (4.1) | 〇Medical cost and monetary compensation should be compensated, calculated based on the other governmental system of compensating injuries4. (4-1-3) | 12 |
| **Procedures and arbitration** |  |  |  |
| Explanation to volunteers at the time of informed consent. | △Company should encourage the investigator to make clear to the participating volunteers that the trial is being conducted in accordance with the guidelines and that copy of the guidelines should be made available when requested. (5.4) | 〇Details of the compensation system should be explained to the volunteer using an outlined paper which should be provided to the volunteer along with the informed consent documents. (4-2) | 12 (See footnote *2) |
| Procedures for management of claims. | 〇Volunteer should claim to the company via the investigator, providing an authority for the company to review medical record. Company should consider the claim expeditiously. (5.1) | 〇Payment for medical expense and medical allowance shall start promptly to relieve volunteer and payment for monetary compensation shall be discussed for assessment of the causal relationship at a committee. (5-2,3) | 9 |
| Dispute resolution. | 〇Independent expert(s) mutually acceptable to the volunteer and the sponsor may be consulted in case of any controversy relating to the level of compensation. (4.3) | 〇Independent expert(s) mutually agreed to by the volunteer and the sponsor may be consulted in case of any controversy relating to the causal relationship or the severity of injury or disability. (6-1) | 7 |

〇 compensation is possible △ case by case × not to be compensated (For the items in “Procedures and arbitration”: 〇 enough △ partially enough × not enough)

*1: In this table, the wording is not exactly the same and the contents do not cover all of that in both guidelines, but the authors outlined in simple manner. ABPI has separate compensation guidelines for patients and for healthy volunteers, whereas JPILA includes both in one guideline, making difference between these two areas. Because our article focuses on compensation for patient volunteers, we compared these two guidelines focusing on policies relating to compensation for patient volunteers, while adding some specific issues relating to compensation for healthy volunteers in the footnotes.

*2: Of the 68 member companies of the Japan Pharmaceutical Manufacturers Association (JPMA) to whom the questionnaire survey was delivered, 44 companies responded. Of these 44 companies, 12 companies provided documents outlining their policy on compensation, which they provide to volunteers along with the informed consent documents (ICDs).

*3: In Japan, “Adverse Drug Reaction Relief System (ADR Relief System)” for marketed drug excludes carcinostatic substances, and immunosuppressive agents. The 2009 revision of JPILA guidelines states that the compensation policy for adverse reactions of carcinostatic substances should be defined in each protocol based on a benefit/risk assessment.

*4 JPILA guidelines suggest that the amount of compensation for patient volunteers should be calculated based on the rules of the “ADR Relief System” which is the system for compensating injuries caused by marketed drugs. Listed below are the amounts of compensation for 2009 fiscal year (This is because our questionnaire survey of the industry part was of the protocols competed this fiscal year. These amounts may be changed each year considering environmental changes, such as price index or market interest rates.)

For healthy volunteers, the amount of compensation for treatment of injury is the same as those for patient volunteers. However, in cases where health insurance is not available, e.g., in the cases of injuries at the phase 1 clinic, sponsor has to pay 100% of the medical cost. The amount of monetary compensation for the cases of death or permanent disability is calculated based on the rules of the “Law for Workmen’s Compensation” or the “Relief System for Injury to Health with Vaccination”, which provides compensation amounts higher than those provided under the compensation system for patient volunteers (for example, in the case of “category 1” injury shown below, the difference is approximately 1.8 times compared to the case of patient volunteer.)

Under the “ADR Relief System”, the amount of monetary compensation is calculated based on the post-injury classifications. For patient volunteers, compensation is limited to category 1 and 2 injuries, where ordinary life is substantially limited or more seriously affected. For healthy volunteers, almost all irreversible injuries are compensated, from category 1 to 14 injuries defined in the “Law for Workmen’s Compensation” or category 1 to 5 defined in the “Relief System for Injury to Health with Vaccination”.

**Compensation for medical cost under the “ADR Relief System”**

- Medical expenses: The amount of patient’s copay part which is 30% of the total medical expense for necessary medical care, excluding the 70% which is covered by the public health insurance (In case health insurance is not available, sponsor shall pay 100% of the medical cost).
- Medical allowance: For miscellaneous expenditures related to medical care or hospitalization, such as transportation expenses, incidental costs, etc..

(1) Volunteers who receive medical care for three or more days in a month (in cases when patients cannot be in the hospital because of some reason) shall be paid JPY (Japanese Yen) 35,800 per month, and those who receive less than three days medical care in a month shall be paid JPY 33,800 per month.

(2) Volunteers who have been hospitalized for eight or more days in a month shall be paid JPY 35,800 per month and those who are hospitalized for less than eight days in a month shall be paid JPY 33,800 per month.

(3) Those who need both medical care and hospitalization shall be paid JPY 35,800 per month.

**Monetary compensation for disability or death under the “ADR Relief System”**

(JPILA guidelines suggest that the following pension defined under the “ADR Relief System” should be paid in full by multiplying it with the present value coefficient, Leibniz’s coefficient corresponding to the each period (******), based on legally permissible interest rate (5%) at the time of the occurrence of the injury:)

- Disability pension:

(1) category 1: JPY 2,720,400 annually (JPY 226,700 per month~~ly~~)

(2) category 2: JPY 2,175,600 annually (JPY 181,300 per month~~ly~~)

(** mean life expectancy.)

- Pension for raising children handicapped by a medical injury

(1) category l: JPY 850,800 annually (JPY 56,700 per month)

(2) category 2: JPY 680,400 annually (JPY 56,700 per month)

(**The years until the child becomes an adult (18 years old). When the child becomes an adult (18 years old), monetary compensation for disability shall be paid in full by multiplying disability pension with the present value coefficient corresponding to the mean life expectancy.)

- Payments in cases of death:

(1) Bereaved family pension due to death of a volunteer who is a breadwinner in the family:

JY 2,378,400 annually (JY 198,200 per month), up to 10 years

(2) Lump-sum benefits for the bereaved family due to death of a volunteer who is not a breadwinner in the family: JY 7,135,200

(3) Funeral expenses: JY 201,000

(**(1) should be lump-sum payment corresponding to ten years; (2) should be paid same as in the case of “ADR Relief System”; (3) should be paid same as that above, whether or not the volunteer patient is the breadwinner in the family.)
