# Supplementary material for: High Rate of Awarding Compensation for Claims of Injuries Related to Clinical Trials by Pharmaceutical Companies in Japan: A Questionnaire Survey
Source: PLoS One. 2014 Jan 8;9(1):e84998. doi: 10.1371/journal.pone.0084998 (PMC3885663; doi:10.1371/journal.pone.0084998)
Supplement: Table S2 — Expression and impression of the volunteers who received compensation. (DOCX) [file pone.0084998.s002.docx]

**Table S2.** Expression and impression of the volunteers who received compensation

| No. | Who made the claim? | Who explained? | The explanation was easy to understand? | How was the procedure? | How was the content of the compensation? |
| --- | --- | --- | --- | --- | --- |
| 1 | Volunteer | CRC | Yes | Smooth | Enough and satisfactory |
| 2 | Doctor, CRC | CRC | Yes | Smooth | Enough and satisfactory |
| 3 | Doctor, CRC | CRC | Yes | Smooth | Did not match the level of injury |
