# Supplementary figures and images for: High Rate of Awarding Compensation for Claims of Injuries Related to Clinical Trials by Pharmaceutical Companies in Japan: A Questionnaire Survey
Source: PLoS One. 2014 Jan 8;9(1):e84998. doi: 10.1371/journal.pone.0084998 (PMC3885663; doi:10.1371/journal.pone.0084998)

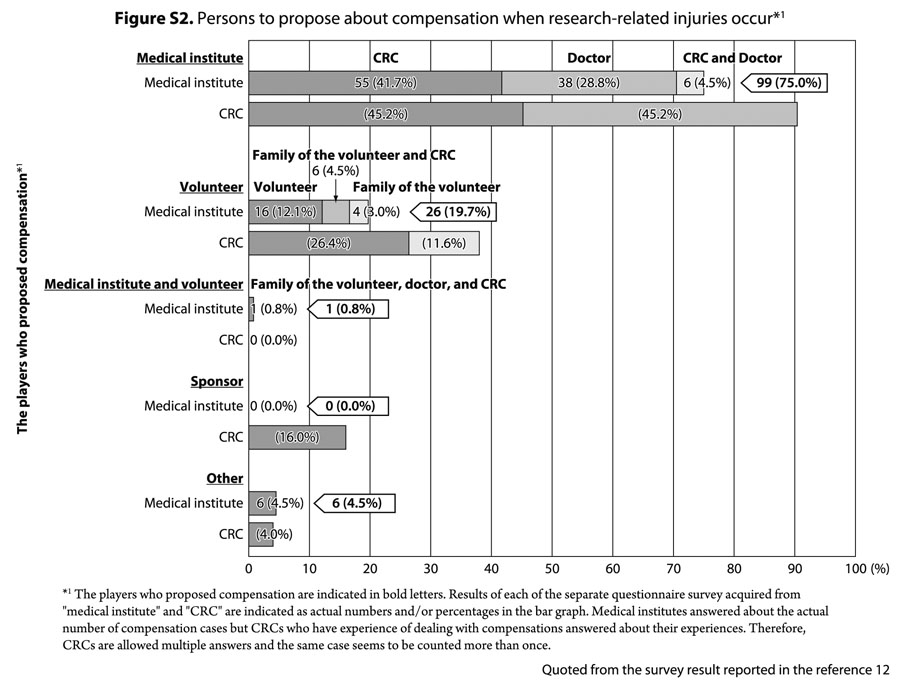

Supplement: Figure S1 — Persons to propose about compensation when research-related injuries occur. (JPG) [file pone.0084998.s003.jpg]
